# Evaluation of eye irritation by S-(-)-10,11-dihydroxyfarnesic acid methyl ester secreted by *Beauveria bassiana* CS1029

**DOI:** 10.3892/etm.2013.1249

**Published:** 2013-08-05

**Authors:** HYEONG-U SON, SANG-HAN LEE

**Affiliations:** 1 Department of Food Science and Biotechnology, Kyungpook National University, Daegu 702-701, Republic of Korea; 2 Food and Bio-Industry Research Institute, Kyungpook National University, Daegu 702-701, Republic of Korea

**Keywords:** *Beauveria bassiana*, eye irritation, toxicity, cosmetic ingredient, dihydroxyfarnesic acid

## Abstract

The aim of this study was to investigate whether S-(-)-10,11-dihydroxyfarnesic acid methyl ester produced by cell subtype *Beauveria bassiana* CS1029 causes acute toxicity when used for cosmetic purposes by performing an eye irritation test. New Zealand white (NZW) rabbits were treated with a 100 mg/dose of S-(-)-10,11-dihydroxyfarnesic acid methyl ester according to standard procedure guidelines. No significant changes in terms of ocular lesions of the cornea, turbidity of the cornea, swelling of the eyelid or ocular discharge were observed in the methyl ester-treated groups, while sodium dioctyl sulfosuccinate, a positive control, caused severe toxicity. The anatomical and pathological observations indicate that the methyl ester produced by *Beauveria bassiana* CS1029 did not induce eye irritation in the lenses of the rabbits. The data suggest that the methyl ester evaluated in this study has promising potential as a cosmetic ingredient that does not irritate the eye.

## Introduction

*Beauveria bassiana* is a type of entomopathogenic fungi used as an insecticide (1). In humans, this fungus has limited toxicity. To the best of our knowledge, only a few cases of keratitis due to Beauveria bassiana have been documented (1). Furthermore, entomopathogenic fungi are known for their beneficial activities in various biological fields (2). Previously, entomopathogenic fungi, including *Beauveria bassiana*, *Cordyceps sinensis, Cordyceps militaris* and *Paecilomyces*, have been used to treat atopic dermatitis, athlete’s foot and dandruff (3). These fungi have also been shown to possess immunomodulatory, anti-diabetic, anti-stress and anti-tumor activities (4); however, their cosmeceutical properties are not adequate for use. Studies concerning the whitening effects of fungal fermentation products have since been initiated (5).

It is considered important to conduct eye irritancy, skin irritancy and phototoxicity tests prior to obtaining approval and authorization for the use of test compounds as functional cosmetic ingredients. Since Draize developed a method for the measurement of irritancy and toxicity of substances applied topically to the skin and mucous membranes (6), numerous trials have been performed to assess the cosmetic and cosmeceuticals effect of various products. However, alternative testing methods, including an *in vitro* 3T3 NRU phototoxicity test and local lymph node assays (7), are increasingly being considered as credible alternatives to animal models for evaluating functional cosmetic ingredients, due to ethical concerns about animal use. A number of potential applications are emerging for the use of biochemicals present in insect extracts (or fractions thereof) as cosmetics/cosmeceuticals. For this reason, we performed toxicity tests to investigate whether S-(-)-10,11-dihydroxyfarnesic acid methyl ester, a compound isolated from *Beauveria bassiana* CS1029, has the potential to induce irritation of the ocular mucosa. To the best of our knowledge, it has not been documented whether insect extracts cause toxicity when the skin or eye lens is exposed to them. Unwanted reactions to cosmetics are frequent in patients with allergic contact dermatitis. Various adverse effects, including acute/chronic toxicity, irritation and sensitization, have been assessed using *in vivo*, *in vitro*, semi *in vivo* and *ex vivo* animal models (8–10).

In the present study, we performed the eye irritation test with a derivative of dihydroxyfarnesic acid produced by *Beauveria bassiana* CS1029 using an *in vivo* animal model. Various parameters were assessed to evaluate the degree of eye irritation induced by the compound and determine whether it is safe for development in cosmetic/cosmeceutical applications.

## Materials and methods

### Animals and care

New Zealand white (NZW) rabbits (9-week-old males weighing 2.1–2.4 kg) were purchased from Samtaco (Osan, Korea). The animals were fed a commercial diet (Purina, Seoul, Korea) and water *ad libitum* throughout the experiment. The study protocols complied with the guidelines of the International Association for the Study of Pain Committee for Research and Ethical Issues (11) and the internal guidelines of the Kyungpook National University Animal Ethical Committee were strictly observed. All animals acclimated to the laboratory environment for at least 1 week prior to commencement of the experiment.

### Isolation and preparation of S-(-)-10,11-dihydroxyfarnesic acid methyl ester

S-(-)-10,11-dihydroxyfarnesic acid methyl ester was produced by *Beauveria bassiana* CS1029. In brief, a fermentation medium consisting of 3% sucrose, 2% corn steep liquor (C4648; Sigma, St. Louis, MO, USA), 0.05% potassium phosphate dibasic, 0.1% potassium phosphate monobasic and 0.05% magnesium sulfate•6H*_2_*O was prepared in a 5-liter mini jar fermenter (Hankook Fermenter, Seoul, Korea). The medium was then sterilized at 121°C for 30 min and chilled prior to inoculation with the seed culture of *Beauveria bassiana* CS1029 up to 5%. Fermentation was performed for 3 days. The fermentation broth was then centrifuged at 10,000 × g for 30 min and the supernatant was added to the following columns as previously described (12). Briefly, the precipitate was applied to an HP chromatography column and high-performance liquid chromatography (HPLC) was performed using a reverse column (Waters, Milford, MA, USA) with a detector at 254 nm (Waters 2998 Photodiode Array detector). A peak was identified as S-(-)-10,11-dihydroxyfarnesic acid methyl ester by nuclear magnetic resonance (NMR) and mass spectroscopy as previously described (12). A voucher specimen (#2009-Bb) of the methyl ester obtained from *Beauveria bassiana* CS1029 was deposited in the Laboratory of Food Enzyme Biotechnology, Kyungpook National University, Korea.

### Eye irritation test

S-(-)-10,11-dihydroxyfarnesic acid methyl ester (100 mg/100 *μ*l) was dripped into the eyes of each NZW rabbit (n=3) which were held open with clips at the lid. As a positive control, 10% sodium dioctyl sulfosuccinate solution was applied. Progressive damage to the rabbit eye was monitored every day for 5 days. Potential reactions to the methyl ester included swelling of the eyelid, iris inflammation, ulceration and hemorrhage (13,14). In brief, the eye lens mucosa was assessed for localized irritation. Saline was used as the control. The conjunctival sac in the right eye of each rabbit was treated with the undiluted methyl ester (0.1 ml), negative control (saline) or positive control (10% sodium dioctyl sulfosuccinate). After applying the solutions once for 2 sec, the eyes were washed with saline. The undiluted methyl ester (0.1 ml) was administered once under the eyelid, which was slightly pulled away from the eyeball to form a space to allow easy delivery into the conjunctival sac. The cornea, iris and conjunctiva were then examined daily (for 1, 2, 3, 4 and 5 days) to evaluate acute irritation of the lens mucosa.

### Analysis of irritancy

The development of eye lesions was monitored by comparison of the treated eye with the left eye that was not treated with the test substance, as previously described (15). On days 1, 2, 3, 4 and 5 after application of the methyl ester or positive control, the following variables were evaluated with the naked eye: corneal opacity and turbidity, reaction of the iris, conjunctival edema and ocular discharge. Irritation of the eye lens mucosa was evaluated based on redness or ocular lesion development by clinical examiners under the direction of a veterinarian from the Center of Laboratory Animal and Care, Kyungpook National University, Korea.

## Results and Discussion

While screening natural resources for active components exhibiting whitening activities that may be used in cosmetics, we identified that *Beauveria bassiana* CS1029 produces a potent compound into the medium during liquid culturing. The compound was identified to be S-(-)-10,11-dihydroxyfarnesic acid methyl ester and was observed to display anti-tyrosinase activity *in vitro* and *in vivo* (12) (data not shown). We subsequently determined whether the agent was capable of ameliorating conditions associated with skin inflammation, including atopic dermatitis (12).

Biomaterials derived from insects and insect-symbiotic fungi may be obtained using a variety of methods, including supercritical extraction, microbial fermentation, biotransformation and chemical modification. Certain biomaterials may be converted into cosmetic, cosmeceutical or neutraceutical ingredients. This prompted our investigation in which we investigated an anti-tyrosinase agent derived from medicinal insect extracts and identified that it exhibited a potent whitening activity (12). To determine whether the agent was toxic or non-toxic and suitable to serve as a cosmetic ingredient, we performed an acute toxicity investigation.

**Figure 1.**
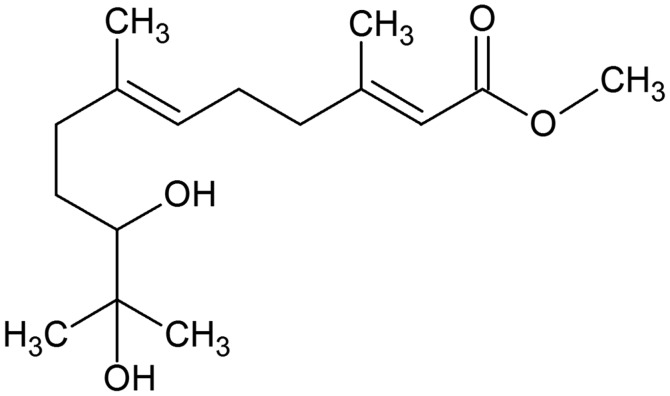
Structure of the S-(-)-10,11-dihydroxyfarnesic acid methyl ester obtained from *Beauveria bassiana* CS1029.

In the present study, S-(-)-10,11-dihydroxyfarnesic acid methyl ester (Fig. 1; final concentration, 100 mg/100 *μ*l) was administered to rabbit eyes and eye irritation data was obtained to determine whether the compound is safe to use. When saline was administered as the control, no adverse symptoms were observed around the pupil or whites of the eye (data not shown). The Draize eye irritation test used in the current study is strictly observational and is not considered to reflect the degree of irritation in humans adequately (13). This technique is, therefore, generally considered crude, imprecise and unreliable; however, it is inexpensive, time-saving and produces potentially convincing data. A number of scientists are seeking alternative testing methods to avoid animal ethics issues.

**Figure 2.**
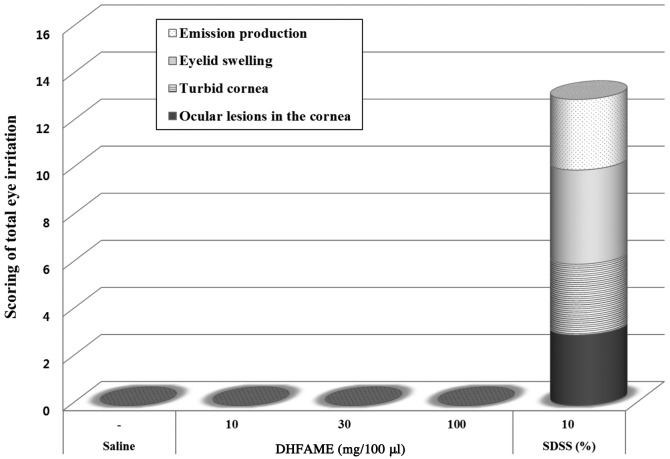
Eye irritation following exposure to the dihydroxyfarnesic acid derivative DHFAME produced by *Beauveria bassiana* CS1029. Final scores of acute eye lens mucosal irritation are shown. Each score represents the sum of each symptom. Data shown are representative results from five independent observations. SDSS, sodium dioctyl sulfosuccinate; DHFAME, S-(-)-10,11-dihydroxyfarnesic acid methyl ester.

In the present study, we precisely evaluated the symptoms of toxicity using the following criteria: swelling, inflammation and lesions on the eye lens. Initially, 24 h after treatment with the methyl ester, morphological changes of the eyelid and ocular mucosa membranes were examined. Ocular lesions in the cornea (represented by the black section in Fig. 2) were scored as follows: 0, no suppuration or haze; 1, slight opacity compared with normal transparency; 2, semi-transparent; 3, no observation at the end of the pupil size; and 4, an opaque and turbid cornea but unaffected iris. The methyl ester did not induce ocular lesions in the cornea (Fig. 2) or any other symptoms (similar to saline). Scores for turbid cornea size (lined section in Fig. 2) were as follows: 0, no turbidity; 1, 1/4 or less; 2, greater than 1/4 to no more than 1/2; 3, greater than 1/2 to no more than 3/4 in size; and 4, greater than 3/4 to the entire cornea affected. The turbid cornea size was not affected by the methyl ester (Fig. 2). The effects of the methyl ester on eyelid swelling were also examined. Eyelid swelling (grey section in Fig. 2) was scored as follows: 0, no swelling; 1, slightly swollen; 2, significant swelling of the eyelid resulting in partial abduction; 3, swelling affecting approximately half of the eyelid; and 4, more than half of the eyelid is swollen. Using this scoring system, we confirmed that eyelids treated with the methyl ester were not significantly swollen (Fig. 2). Production of ocular discharge (dotted section in Fig. 2) was analyzed according to the following scale: 0, no discharge; 1, a small amount of moistness around the eyelashes; 2, wet discharge; and 3, a large area around the eye, eyelid and/or eyelashes containing wet discharge. No discharge was observed in the eye or eyelid/eyelashes following treatment with the methyl ester (Fig. 2). Overall, we did not detect any changes or damage induced by the methyl ester; this was in contrast with sodium dioctyl sulfosuccinate, which caused severe symptoms of toxicity (Fig. 2) based on our clinical observations.

**Figure 3.**
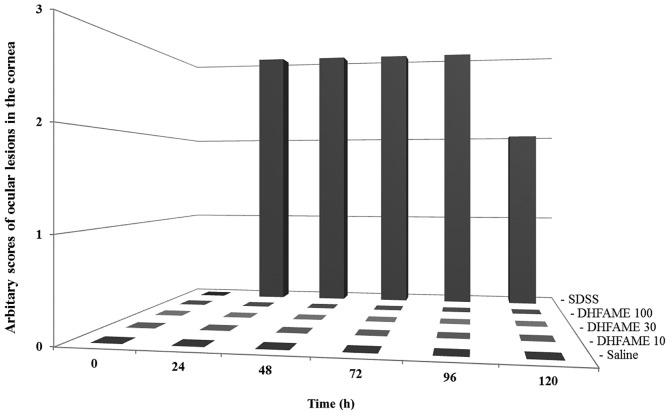
Development of ocular lesions in the cornea due to acute eye irritation over time. Scoring standards are described in Materials and methods. Observations were made at 0, 24, 48, 72, 96 and 120 h after treatment with various reagents. Arbitrary scores for measuring eye irritation (assessed by ocular lesions in the cornea) over time are shown. Data shown are representative results of five independent observations. SDSS, sodium dioctyl sulfosuccinate. DHFAME, S-(-)-10,11-dihydroxyfarnesic acid methyl ester. Lane 1, saline; lane 2, DHFAME (10 mg/100 *μ*l); lane 3, DHFAME (30 mg/100 *μ*l); lane 4, DHFAME (100 mg/100 *μ*l); lane 5, SDSS.

The criteria for determining whether other parameters are associated with acute eye irritation were also assessed. Observations made at different time intervals (day 1, 2, 3, 4 and 5) demonstrated that the methyl ester did not lead to the development of ocular lesions in the cornea (Fig. 3), increase the turbid cornea size (data not shown), eyelid swelling (data not shown) or emission production (data not shown), whereas sodium dioctyl sulfosuccinate produced severe effects (Fig. 3) on days 1, 2, 3, 4 and 5.

Certain toxic effects may be revealed by other safety tests; therefore, we are unable to exclude the possibility of potential toxicity based on acute, sub-acute or chronic safety tests (16–18).

In summary, the dihydroxyfarnesic acid methyl ester produced by *Beauveria bassiana* CS1029 did not induce symptoms of acute eye irritation (haze, swelling, redness or discharge from the ocular lens mucosa) in rabbits. This compound may therefore be suitable for use in the development of cosmetic or cosmeceutical products. Additionally, no toxic effects were observed in the eye irritation test. Future studies using alternative methods to assess eye and skin irritation, as well as phototoxicity *in vitro* and *in vivo*, are required to more fully understand the long-term effects and safety of this compound for used in cosmetics.

## References

[1] Figueira L, Pinheiro D, Moreira R, Pinto E, Simões J, Camisa E, Torrão L, Palmares J, Falcão-Reis F (2012). *Beauveria bassiana* keratitis in bullous keratopathy: antifungal sensitivity testing and management. Eur J Ophthalmol.

[2] Pedrini N, Crespo R, Juárez MP (2007). Biochemistry of insect epicuticle degradation by entomopathogenic fungi. Comp Biochem Physiol C Toxicol Pharmacol.

[3] Zhou X, Gong Z, Su Y, Lin J, Tang K (2009). Cordyceps fungi: natural products, pharmacological functions and developmental products. J Pharm Pharmacol.

[4] Tomoda H, Doi T (2008). Discovery and combinatorial synthesis of fungal metabolites beauveriolides, novel antiatherosclerotic agents. Acc Chem Res.

[5] Liu L, Liu Y, Li J, Du G, Chen J (2011). Microbial production of hyaluronic acid: current state, challenges, and perspectives. Microb Cell Fact.

[6] Draize JH (1959). Appraisal of the Safety of Chemicals in Foods, drugs and cosmetics.

[7] Nigam PK (2009). Adverse reactions to cosmetics and methods of testing. Indian J Dermatol Venereol Leprol.

[8] Tavaszi J, Budai P, Pálovics A, Kismányoki A (2008). An alternative test battery in detecting ocular irritancy of agrochemicals. Commun Agric Appl Biol Sci.

[9] Scott L, Eskes C, Hoffmann S, Adriaens E (2010). A proposed eye irritation testing strategy to reduce and replace in vivo studies using bottom-up and top-down approaches. Toxicol In vitro.

[10] Osborne R, Perkins MA, Roberts DA (1995). Development and intralaboratory evaluation of an in vitro human cell-based test to aid ocular irritancy assessments. Fundam Appl Toxicol.

[11] Zimmermann M (1983). Ethical guidelines for investigations of experimental pain in conscious animals. Pain.

[12] Nam SH, Yoon CS, Jeon JY, Lee SH, Lee KG, Yeo JH, Hwang JS Composition exhibiting melanin-inhibiting activity.

[13] Draize JH, Woodard G, Calvery HO (1944). Methods for the study of irritation and toxicity of substances applied topically to the skin and mucous membranes. J Pharmacol Exp Ther.

[14] Aoshima H, Saitoh Y, Ito S, Yamana S, Miwa N (2009). Safety evaluation of highly purified fullernenes (HPFs): based on screening of eye and skin damage. J Toxicol Sci.

[15] Son HU, Yoon EK, Cha YS, Kim MA, Shin YK, Kim JM, Choi YH, Lee SH (2012). Comparison of the toxicity of aqueous and ethanol fraction of *Angelica keiskei* leaf using the eye irritancy test. Exp Ther Med.

[16] Korting HC, Herzinger T, Hartinger A, Kerscher M, Angerpointner T, Maibach HI (1994). Discrimination of the irritancy potential of surfactants in vitro by two cytotoxicity assays using normal human keratinocytes, HaCaT cells and 3T3 mouse fibro-blasts: correlation with in vivo data from a soap chamber assay. J Dermatol Sci.

[17] Tardiff RG, Hubner RP, Graves CG (2003). Harmonization of thresholds for primary skin irritation from results of human repeated insult patch tests and laboratory animal skin irritation tests. J Appl Toxicol.

[18] Basketter DA, Kimber I (2011). Skin irritation, false positives and the local lymph node assay: a guideline issue?. Regul Toxicol Pharmacol.

